# A soil fumigant increases American ginseng (*Panax quinquefolius* L.) survival and growth under continuous cropping by affecting soil microbiome assembly: a 4-year *in situ* field experiment

**DOI:** 10.1128/spectrum.01757-23

**Published:** 2023-12-15

**Authors:** Na Peng, Yanmeng Bi, Xiaolin Jiao, Ximei Zhang, Junfei Li, Yi Wang, Shanshan Yang, Ziqi Liu, Weiwei Gao

**Affiliations:** 1 Institute of Medicinal Plant Development, Chinese Academy of Medical Science and Peking Union Medical College, Beijing, China; 2 School of Environmental and Municipal Engineering, Tianjin Chengjian University, Tianjin, China; 3 Biomedicine School, Beijing City University, Beijing, China; USDA - San Joaquin Valley Agricultural Sciences Center, Parlier, California, USA

**Keywords:** American ginseng (*Panax quinquefolius *L.), root rot disease, soil fumigation, disease suppression, rhizosphere microbial community

## Abstract

The replant problem is a widespread challenge in agricultural production and results in significant financial losses. The survival and development of American ginseng (*Panax quinquefolius* L.), a well-known perennial medicinal plant, also typically suffer from replant problems. At present, efficient strategies for this issue and the underlying mechanisms are still lacking. Here, a series of *in situ* field experiments over a 4-year period were conducted to investigate the effectiveness and possible mechanisms of soil fumigation and fungicide in improving American ginseng survival under continuous cropping. During the 4-year ginseng cultivation, the seedling emergence rates in the dazomet fumigation-treated group (DF) decreased from 90.67% to 52.92%, from 78.67% to 0% in the lime sulfur fungicide-treated group (LS), and from 66.67% to 0% in the control. The fresh root weight in DF increased from 0.36 to 28.72 g, from 0.32 to 13.74 g in LS, and from 0.42 to 9.06 g in the control for the first 2 years. However, no ginseng survived during the following 2 years. Moreover, DF significantly decreased root rot disease incidence by 55.55% compared to LS-treated soil and 69.39% compared to non-treated soil. The correlation analysis results showed that both diversity and structure indices of bacteria and fungi were significantly correlated with root biomass and disease incidence in the second year. In summary, DF has presented a significant beneficial effect on American ginseng development. Additionally, the second year is the critical period to shape the soil microbial community, which could have a long-lasting effect on the development of American ginseng.

**IMPORTANCE:**

Numerous reports of soil fumigants and fungicides on annual crops exist; however, it is unclear whether the single application to perennial plants persistently improves plant growth and controls disease or whether it has a long-lasting impact on soil microbes. We found that soil fumigation enhances ginseng growth and suppresses root rot disease by reshaping the soil microbial community. Our findings benefit the agricultural development of ginseng and provide a theoretical basis for the prevention of ginseng diseases.

## INTRODUCTION

The perennial herb known as American ginseng (*Panax quinquefolius* L.) is a member of the Araliaceae family. American ginseng is frequently used in pharmaceutical and healthcare products. Its secondary metabolites, ginsenosides, have shown a wide variety of biological activities, including stabilizing blood sugar, improving heart health, and reducing inflammation and tumor growth (1, 2). American ginseng is mainly grown in the northern regions of China, the USA, and Canada due to its unique and particular habitat preferences. China has become the third-largest exporter country because of the rising demand (3). Additionally, it takes at least 3 years for the tuberous roots to meet acceptable medicinal quality standards (4). However, after the third successive planting of American ginseng within the same field, the root rot disease incidence increases by 50%–80%, which subsequently leads to plant mortality (5). This replant problem rendered extreme difficulties in planting American ginseng in the same field for more than 10 years after completing the initial 4-year production cycle (6). Therefore, strategies are critically needed to resolve the continuous cropping-induced disease and growth inhibition and/or to shorten the recovery time of the cropping field.

Several factors have been reported to be associated with the high incidence of root rot disease and the continuous cropping obstacles, such as soil microbial community imbalances, fungal pathogen accumulation, and the increase in allelopathic autotoxicity of root exudates (5). In fact, other cultivated *Panax* (ginseng) plants, such as *Panax ginseng* and *Panax notoginseng*, also suffer from the replant problem. Several studies have reported a few possible causes of continuous cropping failure. For instance, a study by Dong et al. (7) found that after *Panax notoginseng* cropping, reduced fungal diversity is correlated with increased plant mortality. Additionally, another study found that American ginseng-cultivated soil showed an obvious accumulation of fungal pathogens (such as *Fusarium* and *Ilyonectria*) compared with non-cropping soil (8). Similar to the fungal community changes, the bacterial communities were also significantly altered after continuous plantation in a Korean ginseng study. Specifically, the abundance of Acidobacteria, Betaproteobacteria, Gammaproteobacteria, and Sphingobacteria in the soil of the successive cropping field was significantly different from that of the non-cropping field soil (9). Thus, the imbalance of the microbial community and the enrichment of soilborne pathogens in the cultivated soil might be key factors leading to replant problems in ginseng plants.

In a wide range of environmental and cultivation systems, applying pre-plant soil fumigations and fungicides has been found to effectively control soilborne diseases and promote the productivity of crops (10 – 15). Dazomet (3,5-dimethyl-1,3,5-thiadiazinane-2-thione), which releases ozone-friendly methyl isothiocyanate, is frequently used to disinfect ginseng plantation soil (16). The effects of dazomet on pathogens and plant pathogenic nematodes have been well studied over the last 15 years in tomato and lettuce (17 – 19). Lime sulfur (calcium polysulfide), another plant disease control reagent, has been allowed to be used in organic crop production as an insecticide (including acaricide or mite control) by the USDA National Organic Program (NOP). Previous studies showed that lime sulfur could control pear scab and rust (20) and apple scab and cedar apple rust (21). However, whether these fumigants and fungicides can be adapted to American ginseng and effectively mediate root rot disease and replant problems is still unknown. In addition, numerous studies have reported positive results of using soil fumigants and fungicides on annual crops. However, it is unclear if a single application to perennial plants persistently improves plant growth by controlling disease and if it has a long-lasting impact on the health of the soil microbial community.

In this study, we investigated the disease control potential of pre-plant soil fumigation (dazomet) and fungicide (lime sulfur) treatments applied to American ginseng in the context of 4 years of continuous cropping. We determined the plant growth rate and disease incidence resulting from these treatments. We specifically studied (i) American ginseng growth and the occurrence of root rot during second-round planting with continuous cropping with or without fumigation and fungicide treatments; (ii) the effects of these treatments on the rhizosphere soil properties and microbial communities of American ginseng; and (iii) the relationships between the ginseng phenotypic characteristics, the diversity and structure of microorganisms, and the soil chemical properties under the fumigation and fungicide application.

## RESULTS

### The seedling emergence rate, fresh root weight, and root rot disease index of American ginseng

**Fig 1:**
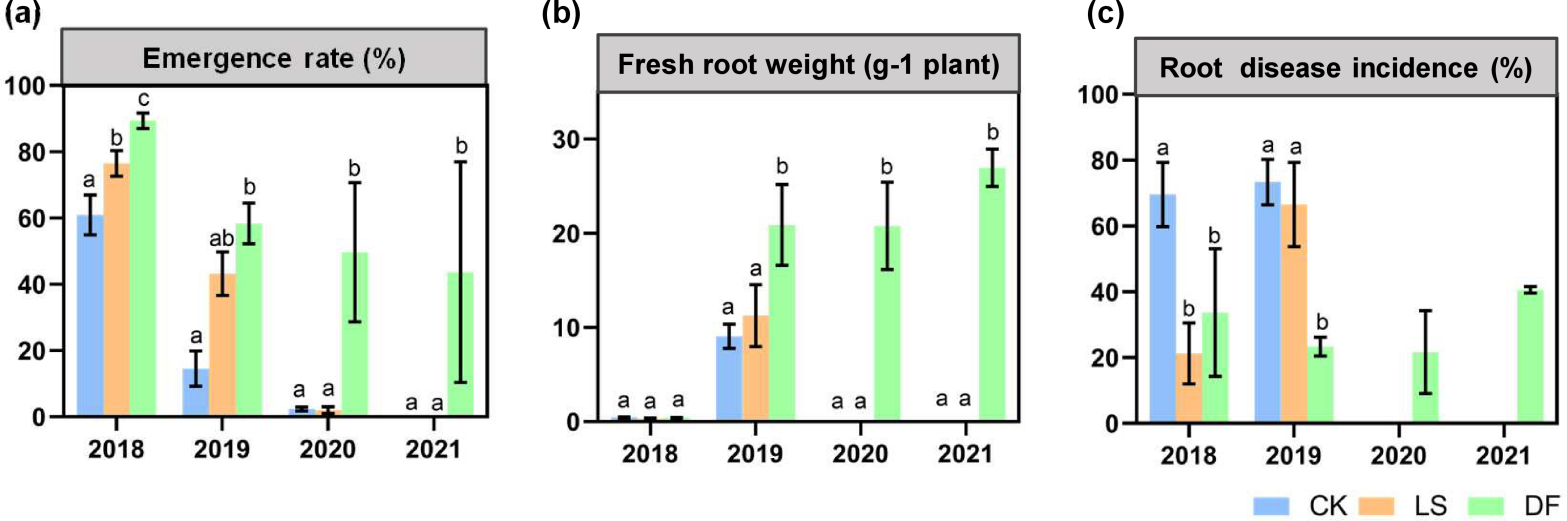
Effect of different soil treatments (CK, without soil fumigant; LS, lime sulfur; DF, dazomet fumigation) on American ginseng phenotypic traits from 2018 to 2021. (**a**) The emergence rate, (**b**) the fresh root weight (biomass), and (**c**) the root disease incidence (DI) of American ginseng plants. Bars above the histogram represent standard deviations, and different letters indicate significant differences (*P* < 0.05) between the different treatments within the year according to ANOVA.

Figure 1 displays the results of soil fumigation or fungicide treatment over a 4-year period on the emergence rate, fresh root weight, and root rot disease index (DI) of American ginseng. In general, a higher emergence rate, higher fresh root weight, and lower DI were observed in the dazomet fumigation (DF) treatment compared with the CK (without soil fumigation) and lime sulfur (LS) treatments.

The emergence rates of American ginseng planted in soil treated with CK, LS, and DF were comparable in 2018. In 2019, compared to the CK treatment, the emergence rate considerably increased with the DF treatment by 301.47% (*P* < 0.001), and the LS treatment raised the emergence rate by 197.06% (*P* < 0.01). In addition, no significant difference was discovered between the LS and DF treatments. In 2020 and 2021, the DF treatment still maintained the emergence rate at 51%–72%. However, the emergence rate of ginseng plants in the CK and LS treatments only ranged from 0% to 2.30% (Fig. 1a).

Furthermore, the effects of soil fumigation or fungicide treatment on the fresh root weight of American ginseng were analyzed (Fig. 1b). In 2018, no significant difference was observed among CK, LS, and DF treatments. In 2019, relative to the CK and LS treatments, the average fresh root weight of ginseng plants in the DF treatment increased by 130.30% (*P* < 0.001) and 85.51% (*P* < 0.001), respectively. Meanwhile, the LS treatment increased the average fresh root weight by 24.14% compared with the CK treatment, but the difference was not significant. The average fresh root weight for the DF treatment was between 21.76 and 28.72 g plant^−1^ over the next 2 years; however, no ginseng plants grew in soils with the LS and CK treatments.

Meanwhile, the effects of these soil fumigation and fungicide treatments on the root rot disease index of ginseng plants were calculated (Fig. 1c). In 2018, relative to the CK treatment, the decrease in DI was significant for the DF treatment (51.55%, *P* < 0.05) and also for the LS treatment (69.39%, *P* < 0.001), but the difference in the disease index between the DF and LS treatments was not significant. In 2019, the DF treatment reduced the disease index by 68.18% (*P* < 0.05) and 64.93% (*P* < 0.05) compared with the CK and LS treatments, respectively. In the following 2 years, the plants grown in soil with the DF treatment demonstrated the least root rot symptoms (DI was 21.67% in 2020 and 40.56% in 2021), while those with the CK and LS treatments had almost no root survival.

### Rhizosphere soil chemical properties

The rhizosphere soil chemical properties are shown in Table S2. In general, no significant differences in soil pH, available nitrogen (AN), available phosphorus (AP), available kalium (AK), and organic matter (OM) were observed among the different soil fumigation treatments per year. Significantly higher soil pH was observed in the second 2 years compared with the first 2 years; however, all soil pH values were under 5.5. Higher concentrations of AN were observed in the first year compared with the following 3 years. The lowest concentration of AP was observed in the first year. Significantly higher concentrations of AK and OM were observed in the first 2 years compared with the second 2 years.

### Microbial community diversity and composition

A total of 462,507 and 468,690 validated bacterial and fungal sequences, respectively, were obtained after passing quality filtering. The number of bacterial and fungal sequence reads per sample ranged from 9,269 to 13,144 [mean ± standard deviation (SD), 12,845 ± 611] and from 12,844 to 13,223 (mean ± SD, 13,016 ± 107), respectively. We detected a total of 1,841 and 471 operational taxonomic units (OTUs) of bacteria and fungi, respectively. The alpha diversity of each sample using the Shannon indices to measure species diversity was analyzed (Fig. S1 and S2). In 2018, the bacterial Shannon index in the DF treatment significantly increased, and its fungal Shannon index significantly decreased as compared to that in the CK treatment, whereas the LS treatment exhibited no discernible difference. In 2019, the fungal Shannon index increased, and the bacterial Shannon index decreased in the DF and LS treatments compared to the CK treatment. In addition, the bacterial and fungal Shannon indices in these three treatments showed no discernible variation during the ensuing 2 years (Fig. S1). However, no annual change in the bacterial and fungal community diversity was seen in DF. Instead, the bacterial community diversity in the CK and LS treatments exhibited a substantial rise over the 4 years, while the fungal community diversity in CK showed a significant decrease (Fig. S2).

**Fig 2:**
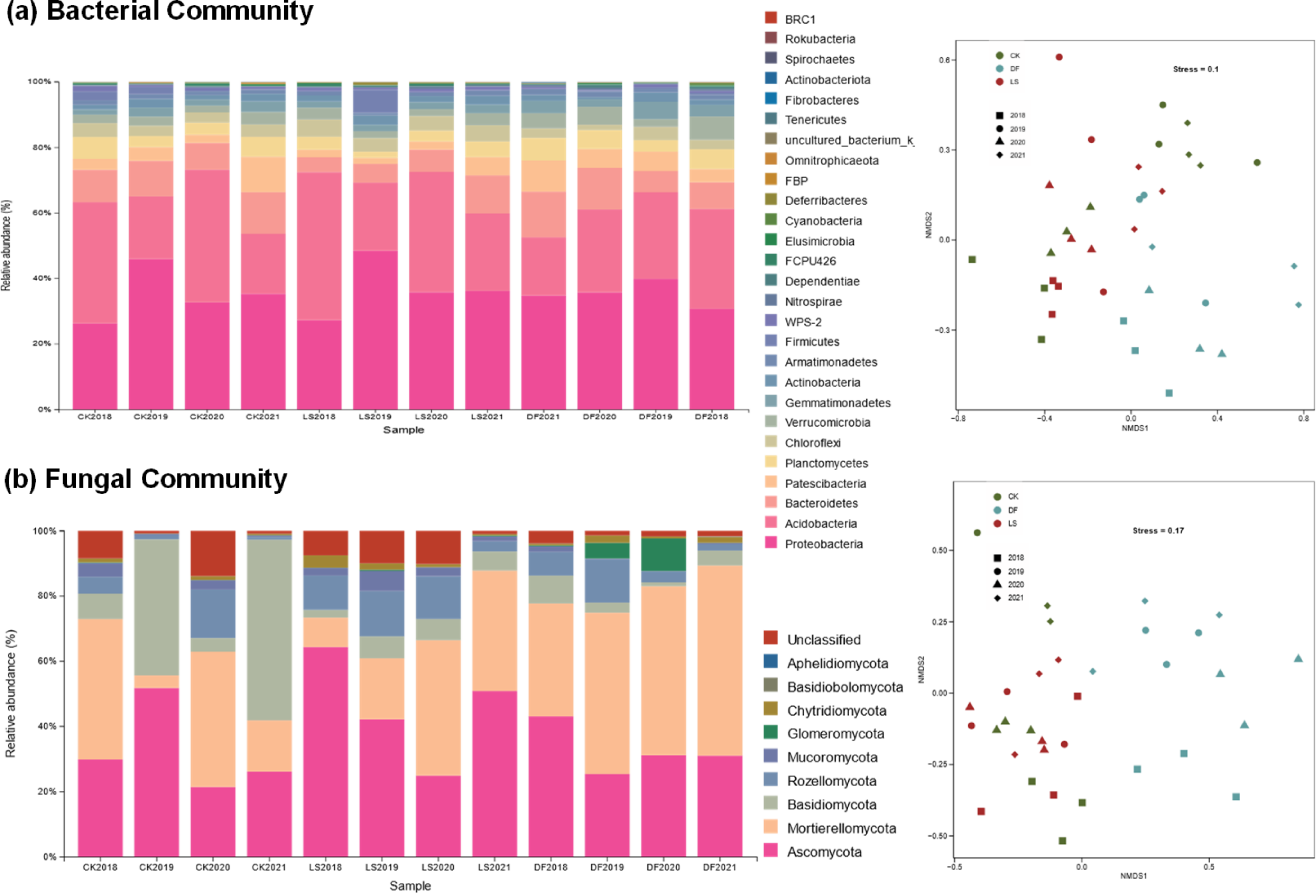
Effect of different soil fumigation treatments (CK, without soil fumigation treatment; LS, lime sulfur treatment; DF, dazomet fumigation treatment) on bacterial and fungal community composition from 2018 to 2021. Nonmetric multidimensional scaling (NMDS) analysis based on the 16S rRNA sequences and the relative abundances of bacterial phyla (**a**) and the ITS sequences and the relative abundances of fungal phyla (**b**).

Furthermore, visual analysis of the nonmetric multidimensional scaling (NMDS) plot showed close clustering of compartments in the first-year plantations, which were distinct from those of the 2-, 3-, or 4-year ginseng cultivation. The rhizosphere soil communities in the DF group were clearly separated from the communities in the CK and LS treatments. At the same time, the principal coordinate analysis (PCoA) revealed differences in bacterial and fungal communities among the three treatments (Fig. S3). For the bacterial community, the cumulative variance explained by the first and second axes (PCoA1 and PCoA2) was 51.88%, 50.87%, 62.13%, and 60.67% in 2018, 2019, 2020, and 2021, respectively (Fig. 2a). For the fungal community, the cumulative variance explained by the first and second axes (PCoA1 and PCoA2) was 46.88%, 75.35%, 70.58%, and 70.92% in 2018, 2019, 2020, and 2021, respectively (Fig. S3b). In PCoA plots, significant spatial variations were observed in the composition of the bacterial and fungal communities among the CK, LS, and DF treatments (*P* < 0.05) (Fig. S3a and b). Additionally, distinct annual variation was observed in the composition of the fungal community but not in DF (*P* = 0.057) (Fig. S3).

In detail, the bacterial sequences were classified into 27 phyla, and the dominant phyla across all samples were Proteobacteria (35.45 ± 7.55%), followed by Acidobacteria (28.43 ± 9.63%), Bacteroidetes (9.76 ± 4.24%), Patescibacteria (4.90 ± 3.20%), Planctomycetes (4.58 ± 1.98%), Chloroflexi (3.63 ± 1.42%), Verrucomicrobia (3.45 ± 1.84%), Gemmatimonadetes (2.63 ± 1.21%), Actinobacteria (1.88 ± 0.88%), Firmicutes (1.61 ± 3.49%), and Armatimonadetes (1.25 ± 0.62%), accounting for 98% of the total bacterial sequences (Fig. 2a). In particular, the DF and LS treatments significantly reduced the relative abundance of Firmicutes by 90.60% (*P* < 0.001) and 92.17% (*P* < 0.001) in 2018, respectively, compared with the CK treatment. However, the relative abundance of Firmicutes was 646.42% (*P* < 0.001) lower in the DF treatment and 231.58% (*P* < 0.001) higher in the LS treatment than in the CK treatment for the second year. By the way, there was no significant difference among the three treatments in the next 2 years.

Unlike the bacterial community, in addition to spatial variations, significant annual changes were observed in fungal communities (*P* < 0.05) (Fig. 2b). In 2018, Ascomycota (29.97 ± 6.87% in CK, 63.65 ± 31.30% in LS, and 43.01 ± 10.74% in DF) was dominant in all the treatments, followed by Mortierellomycota (43.05 ± 8.02%, 9.22 ± 6.34%, and 34.61 ± 15.47%, respectively); however, Basidiomycota accounted for only 7.77 ± 4.08%, 2.43 ± 1.17%, and 8.50 ± 9.93%, respectively. In 2019, the relative abundance of Mortierellomycota (3.89 ± 17.25%) clearly decreased in the CK treatment, whereas the relative abundance of Ascomycota (51.71 ± 18.37%) and Basidiomycota (29.11 ± 24.31%) significantly increased. Instead, in the LS treatment, the relative abundance of Ascomycota (25.14 ± 6.81%) decreased, whereas the relative abundance of Basidiomycota (49.50 ± 2.91%) significantly increased. In the DF treatment, only the relative abundance of Mortierellomycota (49.50 ± 2.91%) increased. In 2020, the relative abundance of Mortierellomycota (41.69 ± 9.36%) significantly increased in the CK treatment, whereas the relative abundance of Ascomycota (21.65 ± 5.11%) and Basidiomycota (4.23 ± 0.55%) significantly decreased, while LS and DF did not change considerably. In 2021, the relative abundance of Mortierellomycota (15.72 ± 5.53%) significantly decreased in the CK treatment, whereas the relative abundance of Basidiomycota (4.23 ± 0.55%) significantly increased. In the LS treatment, the relative abundance of Ascomycota (51.45 ± 1.81%) significantly increased, while that in the DF treatment did not change considerably.

### Relationship between ginseng phenotypic characteristics with rhizosphere microbial community diversity and soil chemical properties

**Fig 3:**
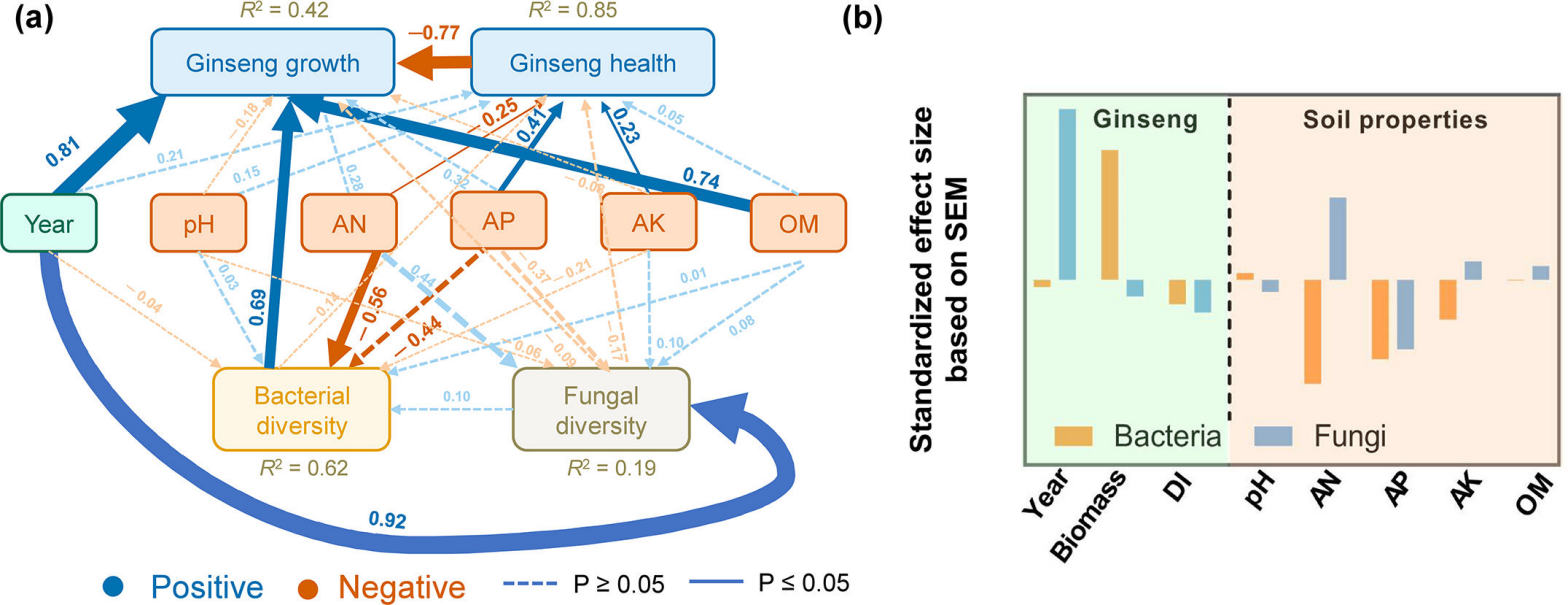
Structural equation models (SEMs) showing the relationships among treatments, soil and plant variables, and bacterial and fungal richness (**a**). Blue and red arrows indicate positive and negative relationships, respectively. Solid or dashed lines indicate significant (*P* < 0.05) or non-significant relationships. Numbers near the pathway arrow indicate the standard path coefficients. *R* ^2^ represents the proportion of variance explained for every dependent variable. *χ* ^2^ = 5.63, d.f. = 2, and *P* = 0.06 (a large *P* value indicates that the predicted model and observed data are equal, that is, good model fitting). Comparative fit index (CFI) = 0.965, and *n* = 36 independent plots. (**b**) Standardized total effects (direct plus indirect effects) derived from SEMs.

To further disentangle the direct and indirect effects of the environmental and planta drivers on microbial biodiversity, structural equation modeling (SEM) analyses were performed (Fig. 3a). Ginseng health (DI) was significantly positively correlated with soil AP and AK (*b* = 0.23–0.41, *P* < 0.05) but significantly negatively correlated with soil AN (*b* =  −0.25, *P* = 0.007). Ginseng growth (biomass), which was significantly positively affected by planting year (*b* = 0.81, *P* = 0.006) and OM (*b*  =  0.74, *P* = 0.005) but significantly negatively affected by ginseng health (DI) (*b*  =  0.45, *P* < 0.001), played the strongest role in directly shaping bacterial richness (*b* = 0.69, *P*  =  0.005; Fig. 3). Soil AN was significantly negatively (*b* = −0.56, *P* = 0.01) correlated with bacterial richness. In comparison, among the variables that directly contributed to fungal richness, only the path of cultivation year (*b* = 0.92, *P* = 0.014) was significant, suggesting that the environmental drivers appear different between bacteria and fungi. Overall, these variables can explain 62% and 19% of the variations in bacterial and fungal richness (Fig. 3a), respectively. In addition, SEM analysis revealed that ginseng properties played predominant roles in shaping microbial diversity (standardized total coefficient  =  0.70 for bacteria and 0.92 for fungi; Fig. 3b) compared with soil properties (standardized total coefficients = −0.56–0.44; Fig. 3b). These results indicate that plant variables are important in mediating rhizosphere soil microbial diversity increases both directly and indirectly.

In the first 2 years, there was a substantial difference in the phenotypic characteristics of American ginseng among all the fumigation and fungicide treatments; however, in the final 2 years, neither the CK nor LS treatments showed any trace of plant growth. To determine the association between ginseng growth and the richness of the microbial community and the chemical characteristics of the soil in the first 2 years, the Spearman correlation analysis was carried out. To confirm the autocorrelation among the ginseng phenotypic traits, a correlation analysis was conducted prior to analysis. A significantly positive and negative correlation was observed between fresh root weight (biomass) and root disease incidence in 2018 (*R* ^2^ = 0.50, *P* < 0.05) and 2019 (*R* ^2^ = 0.62, *P* < 0.05), respectively (Fig. S4). The bacterial community structure indices (*R* ^2^ = 0.83, *P* < 0.001) showed a significantly negative correlation with biomass only in 2019, whereas the fungal community structure indices (*R* ^2^ = 0.45, *P* < 0.05) showed a significantly positive correlation with biomass. However, the biomass of American ginseng showed no correlation with soil nutrients, except for AN (*R* ^2^ = 0.57, *P* < 0.05), which showed a positive correlation in 2019 (Fig. S4). Similar correlations were found between DI with microbial community diversity and soil chemical properties (Fig. S5). No correlation was observed between DI and soil chemical properties. Only fungal community structure was significantly correlated with DI in 2018. The DI was significantly correlated with the community diversity and structure of the bacterial and fungal communities in 2019.

### Microbial indicators of biomass and disease incidence

**Fig 4:**
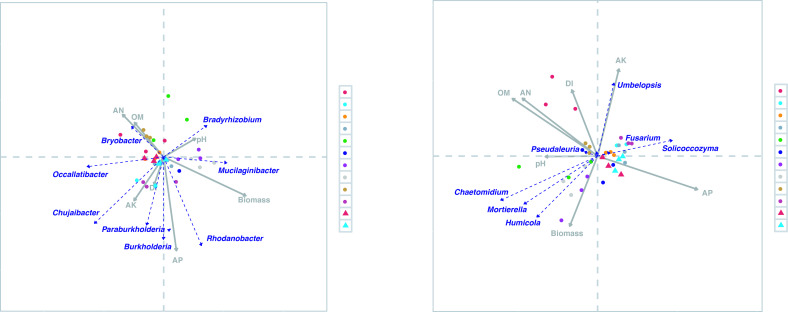
Correlation between bacterial (**a**) and fungal (**b**) communities with fresh root weight (biomass) and root rot disease incidence of American ginseng from 2018 to 2021 explored using redundancy analysis (RDA).

**Fig 5 F5:**
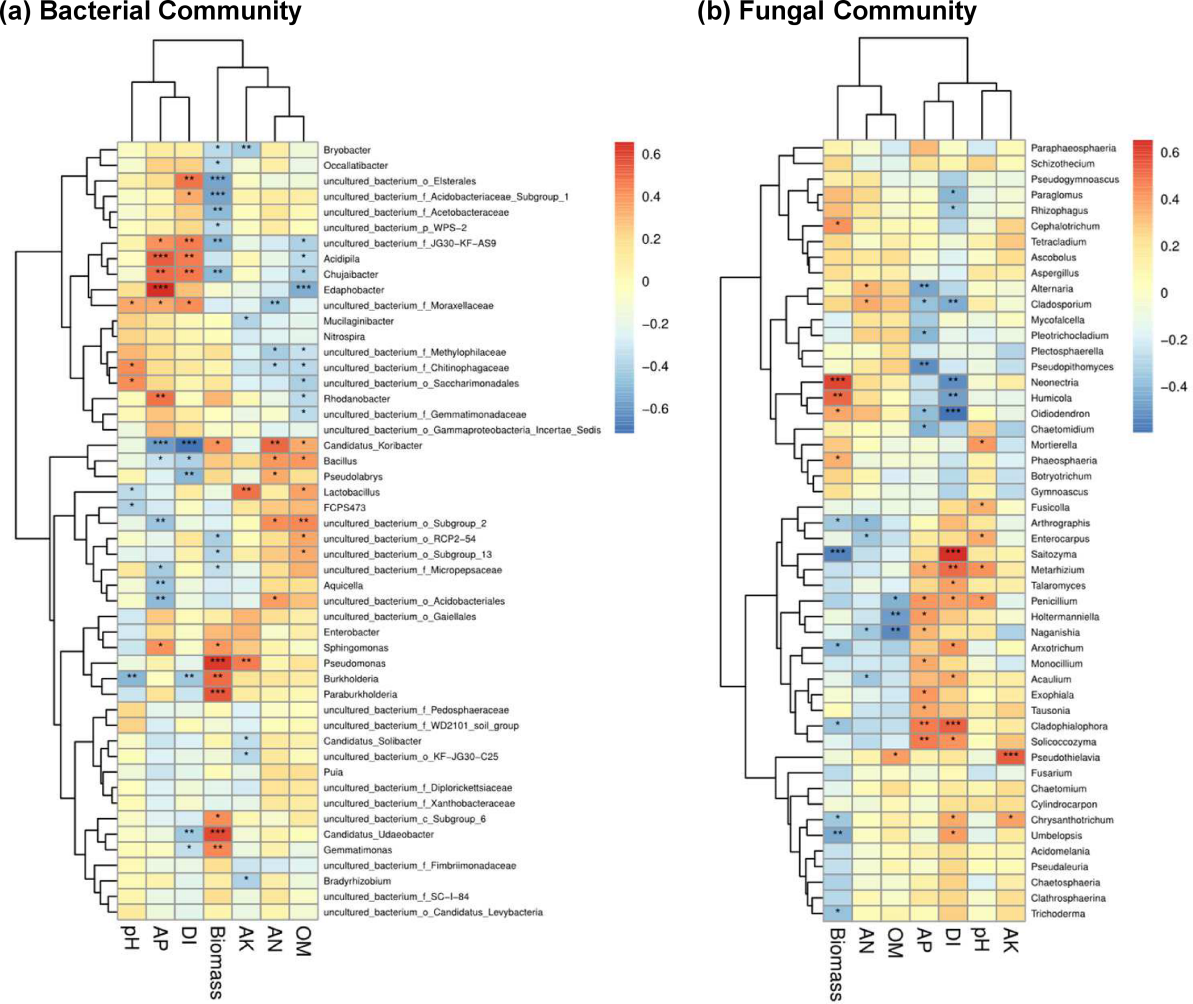
The correlation between pH, AK, AN, OM, AP, fresh root weight (biomass), and root rot disease incidence of American ginseng with the bacterial (**a**) and fungal communities (**b**) at the genus level from 2018 to 2021. Significance levels were presented in the heatmap cell if the correlation was significant (significance levels: *P < 0.05; **P < 0.01; ***P < 0.001).

To find microbial indicators of ginseng biomass and disease incidence from 2018 to 2021, redundancy analysis (RDA) was conducted on microbial communities and ginseng phenotypic characteristics at the genus level (Fig. 5). According to the bacterial communities, most bacteria, such as *Bryobacter*, were negatively correlated with the DI and biomass of American ginseng in 2018. After the second year, the main bacteria were positively correlated with the DI and biomass in the CK and LS treatments but not in the DF treatment (Fig. 4a). With the correlation analysis of the bacterial community and plant growth and health properties from 2018 to 2021 ([Fig F5] Fig. 5a), the results showed that *Bryobacter*, *Occallatibacter*, and *Chujaibacter* were significantly negatively correlated with biomass, while *Candidatus_Koribacter*, *Sphingomonas*, *Pseudomonas*, *Burkholderia*, *Paraburkholderia*, *Candidatus_Udaeobacter*, and *Gemmatimonas* were significantly positively related to biomass. Interestingly, regarding fungal communities, *Umbelopsis*, *Pseudaleuria*, and *Fusarium* were positively correlated with DI but negatively correlated with biomass in the CK and LS treatments during the 4 years of cultivation. In contrast, the composition of the fungal community in soil treated with DF had a higher correlation with *Mortierella*, *Chaetomidium*, and *Humicola*, which were negatively and positively correlated with DI and biomass (Fig. 4b). In particular, *Saitozyma*, *Cladophialophora*, *Chrysanthotrichum*, and *Umbelopsis* were significantly positively correlated with DI but significantly negatively related to biomass (Fig. 5b).

## DISCUSSION

### Soil fumigation promotes American ginseng plant growth and disease control under a continuous cropping system

The current study was conducted in a field that was rotated for 5 years after planting ginseng. The results showed that no ginseng survived either with fungicide treatment or without any treatment after 2 years of continuous cropping. However, the pre-plant soil fumigation treatment applied to American ginseng culture could enhance ginseng growth, suppress root rot disease, and reassemble the soil microbial community. One explanation is that changes in soil chemical parameters could affect the composition of the microbial community (5, 8, 22 – 24), resulting in nutrient acquisition by roots and thus promoting ginseng growth (5). However, the bacterial community, especially its richness in our study, strongly and directly affected fresh root weight and indirectly affected the DI (Fig. 3a). Further, the structure of the soil microbial community was significantly correlated with the ginseng biomass and root rot disease index from the second year (Fig. S4 and S5). Therefore, we inferred that the soil fumigation treatment might promote American ginseng development by reshaping the soil bacterial communities. Meanwhile, the second year is the critical turning point in the formation of the communities.

### Soil fumigation treatment affects rhizosphere microbial diversity

The fungal diversity and bacterial diversity were maintained at a stable level in the soil fumigation treatment; however. the other two treatments showed greater variation (Fig. S1 and S2). Various factors, including plant genotype, growth stage, and others, have been reported to impact microbial diversity (25, 26). In our study, soil samples in all treatments were taken from one American ginseng cultivar at the same growth stage, which was the fruiting stage of ginseng grown over 4 years; thus. the effect of plant genotype on the microbial community could be excluded. Therefore, this result suggested that the main factor impacting rhizosphere microbial diversity was different pre-plant soil treatments. As shown in this work, higher bacterial diversity was found in the fumigation treatment, which produced higher biomass and grew faster. This is consistent with durum wheat (*Triticum turgidum* ssp. durum), which grows more quickly, has less root exudation, and has a higher diversity of bacteria after soil fumigation treatment (27). This may be because these bacteria may improve soil essential nutrition biodynamics and uptake for American ginseng growth (5). On the other hand, fumigation treatment generally presented lower fungal diversity. Previous research has proposed that low soil fungal diversity might be linked to a low soilborne disease (28, 29). These results indicated that the decline in fungal diversity may be a result of a decrease in pathogenic fungi.

### Soil fumigation treatment changes the composition of the rhizosphere microbial community

Moreover, significant differences in the composition of bacterial and fungal communities were found among different soil treatments in the examination of different phyla levels, and the abundance of certain dominant bacterial and fungal groups showed significant differences among the treatments. These findings demonstrated that different soil treatments affected the composition of the rhizosphere microbial community. The different treatments might not result in different specific phyla in the community and may lead to changes in the dominance of only certain phyla in the microbial community. Some microorganisms had a special sensitivity after fumigation or fungicide application, which is in accordance with previous studies showing the effect of soil treatments on rhizosphere communities (18, 28, 29). Furthermore, the composition of the soil bacterial and fungal communities obviously separated among each treatment, as revealed by the nonmetric multidimensional scaling and principal coordinate analysis results regardless of fumigation or fungicide (Fig. 2; Fig. S3). Similar results have been reported in rapeseed meal and broccoli residues (28) and banana monocropping (29). Moreover, soil fumigation altered and modulated the rhizosphere fungal composition (Fig. S3) and then probably induced a reassembly of a non-disease-conducive community from an originally pathogen-dominated community, which may have played an important role in disease control. This speculation was further confirmed by the Spearman correlation results, which showed that the fungal composition index was negatively correlated with the root rot disease incidence and positively related to American ginseng biomass (Fig. S4 and S5).

### Rhizosphere functional microorganisms associated with the plant development of American ginseng

Proteobacteria, Firmicutes, Acidobacteria, Bacteroidetes and Basidiomycota, Ascomycota, and Zygomycota were the most abundant bacterial and fungal phyla across all samples, confirming what was already observed in ginseng-cultured soils (5, 8, 9, 30); however, the relative abundance of phyla was different. Further analysis revealed that the 1-year-old ginseng rhizosphere community was not related to the root rot disease index or root biomass. Instead, there was a significant correlation between bacterial and fungal communities in the second year. This demonstrates that the 2-year-old ginseng rhizosphere microbes may be one of the crucial factors affecting the following survival and development of American ginseng.

For bacteria, the relative abundance of the genus *Chujaibacter* was significantly positively correlated with the disease index and negatively correlated with the growth of American ginseng (Fig. 5), which indicated that certain *Chujaibacter* bacteria may be associated with root rot as well as ginseng mortality. Other functional bacteria, such as *Burkholderia*, *Candidatus_koribacter*, *Pseudomonas*, Paraburkholdera, *Candidatus_Udaeobacter*, and *Gemmatimonas*, had significant positive ginseng growth-promoting effects, which has been reported (31 – 33). In particular, *Bacillus*, which has been reported to secrete many antifungal compounds that protect plants against attack by soilborne pathogens (32, 34, 35), may have the potential ability to inhibit disease occurrence. However, *Pseudomonas* was also significantly correlated with DI, and this may suggest its double-face function of *Pseudomonas* (36).

For fungi, *Mortierella* was the most abundant genus in the rhizosphere soils of all treatments, accounting for 3.89% to 58.63% of the total fungal sequences. Previous studies have suggested that some species of *Mortierella* produce antibiotics, and several isolates have been demonstrated as potential antagonistic agents against various plant pathogens (5, 29, 37). This taxon may potentially serve as a crucial signal of the management of *Fusarium* disease in ginseng farming. Meanwhile, the RDA results showed that *Mortierella* was positively correlated with the root biomass of American ginseng, which suggests that it could be an indicator of American ginseng growth. Another important fungus in American ginseng disease research is *Fusarium*, such as *F. solani* and *F. oxysporum*, which are the main pathogens that can cause severe root rot disease and contribute to significant yield losses for a wide range of medicinal plants (7, 38). Our results showed that *Fusarium* was positively correlated with the disease index but not significantly. This may suggest that as ginseng grows, there are other potential pathogenic microbes that aggravate the occurrence of disease, such as *Cladophialophora* and *Saitozymas*.

Overall, our findings are beneficial to the agricultural development of ginseng and provide a theoretical basis for the prevention of ginseng diseases. In the future cultivation of ginseng, perhaps we can artificially increase or decrease the abundance of certain microorganisms to increase the survival time of cultivated ginseng. As the soil samples were collected from only one ginseng cultivar in our study, our results do not represent the rhizosphere microbial community of *Panax* L. Therefore, the rhizosphere soil from distinct *Panax* L plants should be selected in future research to clearly understand the dynamic changes in *Panax* L rhizosphere microorganisms and provide a scientific basis for the development of the *Panax* L industry.

### Conclusions

In this study, we reported the first targeted tracking investigation of the rhizosphere soil bacterial and fungal communities during American ginseng’s 4-year continuous cultivation. The present study suggested that using dazomet for soil fumigation continuously promotes ginseng growth and suppresses root rot by reshaping the soil microbial community. We also discovered that in addition to root rot-related *Fusarium* fungal pathogens, certain rhizosphere bacteria may be associated with pathogenicity, and the short survival time of American ginseng, including *Lactobacillus*, *Pseudomonas*, and *Burkholderia* and other beneficial fungi, such as *Mortierella*, *Penicillium*, *Monocillium*, and *Naganlshla*, were related to disease suppression. Therefore, to accurately track the roles of soil microbiomes in perennial medicinal plant-microbe interaction, future studies will need to determine dynamic changes in microbial functional genes related to plant diseases temporally and/or spatially to confirm our study’s main conclusions.

## MATERIALS AND METHODS

### Site description and experimental design

**Fig 6:**
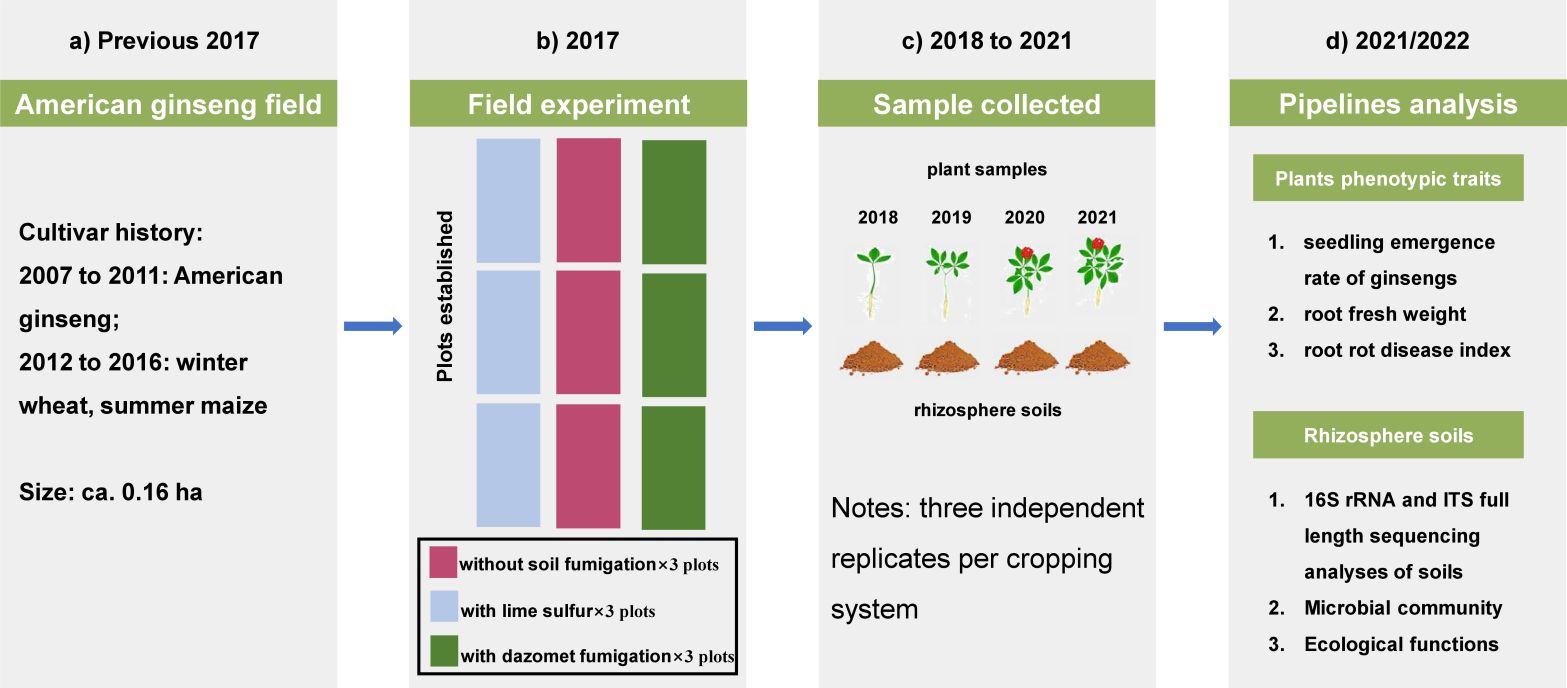
Flow diagram of the key experimental arrangements in the current study. (**a**) Experimental plots were established on a representative American ginseng cultivation field. (**b**) From March 2017, the experimental plots were managed under three cropping treatments. (**c**) At harvest, the plants and rhizosphere soil samples from five pots per field plot were pooled from 2018 to 2021. (**d**) This resulted in three independent replicates for each field cropping system in the subsequent analyses.

The flow diagram of the key experimental arrangements in the current study is shown in Fig. 6. The experimental site is located in Xiguandao Village, Zhangjiazhan Town, Wendeng District, Weihai City, Shandong Province (122°6′42″E, 37°4′21″N), China, with an experimental field area of 0.16 ha. This region is located in the northern temperate zone and has a continental monsoon climate with four distinct seasons. The mean annual precipitation is approximately 767.8 mm, and the annual average temperature is 11.9°C. The experimental field was planted with American ginseng seeds and then grown from 2007 to 2011 and then cultivated with winter wheat and summer maize from 2012 to 2016. After that, American ginseng was planted in 2017 and then grown for 4 years (2017–2021). The soil belongs to the loamy sand soils based on the USDA Soil Survey Manual (Soil Survey Division Staff, 1993). The detailed soil chemical properties before second-round American ginseng cropping are shown in Table S1.

The field experiment was conducted with three replicates for three treatments: continuous cropping of American ginseng (i) without soil fumigation, (ii) with lime sulfur (the original lime sulfur solution was diluted to a Baume degree of 7° and then sprayed in plots at 66.67 kg hm^−2^), and (iii) with dazomet fumigation (dazomet fumigation was applied as a wettable powder with a dosage of 2 kg hm^−2^). The LS and DF treatments were applied once before planting. A ridging pattern of 30 cm was used before planting ginsengs. Each plot was 1.4 m wide ×10 m long, and plots were 20–50 m apart from each other. We used a blue and black sun-shading net to culture American ginseng. Basal fertilizer (125 kg hm^−2^ nitrogen fertilizer, 33 kg hm^−2^ phosphorus fertilizer, and 217 kg hm^−2^ potassium fertilizer) was applied to each plot before ginseng seedlings per year.

### Sample collection

In our study, soil samples in all treatments were taken from one American ginseng cultivar at the same growth stage, which was the fruiting stage of ginseng grown over 4 years; thus, the effect of plant genotype and growth stage on the microbial community could be excluded. American ginseng roots and the corresponding rhizosphere soil were collected in July 2018, July 2019, July 2020, and October 2021, and the months of July to October were the peak period of ginseng root rot disease, which was reported in our previous study (39). A total of 72 soil samples were collected from the same field with varying planting years. The zig-zag random sampling method was adopted (40). The ginseng roots were separated from the soil; the remaining root-adhering soil collected by shaking off from the roots was considered the rhizosphere soil (41, 42). The root tissues were removed and stored for subsequent analysis. Then, the soils were blended thoroughly and put into sterile polythene bags. After the samples were transported to the laboratory, 36 soil samples were stored at −80°C until further use and 36 soil samples were air-dried at room temperature for analysis of physiochemical properties.

### Ginseng seedling emergence rate, root fresh weight, and disease index calculations

The emergence rate (ER) of ginseng was calculated in May 2018, May 2019, May 2020, and May 2021 for each plot. The ER was defined as the number of emerged seedlings in a 3.6-m^2^ area divided by the total number of seedlings. Roots of American ginseng were harvested in July 2018, July 2019, July 2020, and October 2021 to obtain root fresh weight. Rust-colored roots were considered diseased, which have been found among the harvested ginseng. Root rot severity in American ginseng was visually assessed on a 0 to 4 scale as follows: 0 (healthy roots); 1 (<1/10 of the root surface decayed); 2 (1/10–1/3 of the root surface decayed); 3 (1/3–2/3 of the root surface decayed); and 4 (>2/3 of the root surface decayed or the whole plant died). The disease index was calculated as follows:


$$DI=\frac{\sum (Si\times Xi)}{4\times N}\times 100$$

where *Si* is the degree of disease; *Xi* is the number of American ginseng plants with the disease; 4 is the highest degree of disease in the diseased American ginseng plants; and *N* is the total number of American ginseng plants (43).

### Rhizosphere soil chemical properties

The soil samples were mixed with distilled water at a ratio of 1:2.5 to remove CO_2_. After 30 min, the pH was measured using a pH meter (Mettler Toledo, FP20). Soil organic matter was measured via loss-on-ignition at 450°C for 4 h in an oven. Available nitrogen was measured using the alkaline hydrolysis diffusion method, and available phosphorus was measured using the molybdenum-antimony anti-colorimetric method. Available kalium was extracted with ammonium acetate, and its concentration was determined by flame photometry. All soil characteristics are shown in Table S2.

### DNA extraction, PCR amplification, and amplicon sequencing

We used the NucleoSpin 96 Soil kit (740787.2, Macherey-Nagel, Düren, Germany), as per the manufacturer’s protocol, to extract total DNA from rhizosphere soil samples. Furthermore, we tested the integrity, concentration, and purity of DNA using electrophoresis on a 1% agarose gel. The 16S rRNA and ITS genes were amplified with primers 27F:1492R (AGRGTTTGATYNTGGCTCAG/TASGGHTACCTTGTTAS-GACTT) and ITS1F/ITS2 (5′-CTTGGTCATTTAGAGGAAGTAA-3′/5′-GCTGCGT-TCTTCATCGATGC-3′) (44), respectively. PCR amplification was performed in a 20-µL mixture containing 1 µL VnF (10 mM) and 1 µL VnR (10 mM), 1 mL DNA template (approximately 5–50 ng of DNA), 0.4 µL KOD FX Neo (TOYOBO), 10 µL KOD FX Neo Buffer, and 4 µL 2 mM dNTP and then supplemented with 20 µL ddH_2_O. The PCR conditions were as follows: 95°C for 5 min, followed by 30 cycles of 30 s at 95°C, 30 s at 50°C, and 1 min at 72°C, with a final extension at 72°C for 7 min. The products were examined using agarose gel electrophoresis and purified using AMPure XP beads (Beckman Coulter, Inc., Brea, CA, USA), as per the manufacturer’s protocol. The purified PCR products were quantified and homogenized to form a sequencing library, which was then subjected to library quality inspection; after passing the quality inspection, the library was sequenced using the PacBio Sequel System (Biomark Biotechnology Co., Ltd., Beijing, China).

Sequencing data analysis was divided into three steps. First, Lima v1.7.0 software (https://github.com/PacificBiosciences/barcoding/) was used to identify circular consensus sequences (CCSs) through barcodes and obtain barcode-CCS sequence data. Second, the barcode-CCSs were filtered to obtain valid sequences, and third, UCHIME v4.2 software (http://drive5.com/usearch/manual/uchime_algo.html) was used to identify and remove the chimera sequences to obtain the optimization-CCS sequence. Sequence data were clustered into operational taxonomic units at a 97% similarity level using the USEARCH (http://www.drive5.com/usearch/) method. Then, DADA2 software was used to process the long-fragment amplicon CCS to obtain accurate OTUs/ASVs (45). Species annotation and abundance analyses were performed using bacterial and fungal 16S rRNA and ITS data sets from the SILVA database to reveal the species compositions of samples.

### Statistical analyses

Statistical analyses were performed using GraphPad Prism 8 (version 8.0b; GraphPad Software, Inc.). Differences between treatments were statistically analyzed using one-way ANOVA followed by Tukey’s pairwise comparisons test. The results are presented as mean ± standard deviation. Bioinformatics analyses were performed using BMKCloud (www.biocloud.net); principal coordinate analysis and analysis of similarities were performed to determine the differences in microbial community structure; and alpha diversity analysis was performed to study microbial community richness and diversity. We annotated bacterial functions using the FAPROTAX database (46) based on currently available literature on cultivated strains. Finally, redundancy analysis was performed to explore the relationships between microbial communities and soil and plant variables. The Spearman correlations among ginseng root biomass, disease incidence, diversity indices, individual microbial groups, and soil chemical properties were tested in GraphPad Prism 8. The microbial community structure variables came from the first axis of PCA for the microbial variables (47), which explained 24.9%–54.7% of these variables.

### Structural equation modeling

To further discern the direct and indirect effects of the environmental drivers on microbial biodiversity, SEM analyses were performed to examine the relationships among experimental treatments, soil and plant variables, and microbial diversity. To address how year affects ginseng growth and health, we used all the microbial or environmental data with different cultivation years. We first considered a hypothesized conceptual model that included all reasonable pathways. Then, we sequentially eliminated non-significant pathways unless the pathways were biologically informative or added pathways on the basis of the residual correlations. The procedure was repeated until the model showed sufficient fitting, with *P* values of the *χ* ^2^ test >0.05 (that is, the predicted model and observed data are not significantly different) and with root mean square error of approximation (RMSE) <0.08. The SEM-related analysis was performed using the lavaan R package (48).

## Data Availability

Sequence data are available from the authors upon request. The data set reported in this study has been deposited in the NCBI Sequence Read Archive (SRA) database (accession numbers PRJNA899689 and PRJNA899932).

## SUPPLEMENTAL MATERIAL

Supplemental material spectrum.01757-23-s0001.docx Fig. S1 to S5; Tables S1 and S2.

ASM does not own the copyrights to Supplemental Material that may be linked to, or accessed through, an article. The authors have granted ASM a non-exclusive, world-wide license to publish the Supplemental Material files. Please contact the corresponding author directly for reuse.
