# Supplementary material for: A soil fumigant increases American ginseng (Panax quinquefolius L.) survival and growth under continuous cropping by affecting soil microbiome assembly: a 4-year in situ field experiment
Source: Microbiol Spectr. 2023 Dec 15;12(1):e01757-23. doi: 10.1128/spectrum.01757-23 (PMC10783004; doi:10.1128/spectrum.01757-23)
Supplement: Supplemental material — Fig. S1 to S5; Tables S1 and S2. [file spectrum.01757-23-s0001.docx]

A soil fumigant increases American ginseng (*Panax quinquefolius* L.) survival and growth under continuous cropping by affecting soil microbiome assembly: A 4-year *in situ* field experiment

Na Peng^1#^, Yanmeng Bi^1,2#^，Xiaolin Jiao^1,3^，Ximei Zhang^1^, Junfei Li^1^, Yi Wang^1^, Shanshan Yang^1^, Ziqi Liu^1^, Weiwei Gao^1^*

^1^ Institute of Medicinal Plant Development, Chinese Academy of Medical Science and Peking Union Medical College, Beijing, 100193, China

^2^ School of Environmental and Municipal Engineering, Tianjin Chengjian University, Tianjin, 300384, China

^3^ Biomedicine School, Beijing City University, Beijing, 100094, China

**Author information**

Na Peng: 839328163@qq.com

Yanmeng Bi: [bym2019@tcu.edu.cn](mailto:bym2019@tcu.edu.cn)

Xiaolin Jiao: [jiao_1110@163.com](mailto:jiao_1110@163.com)

Ximei Zhang: zhangximei2008@163.com

Junfei Li: ljf114129@163.com

Yi Wang: 18854800636@163.com

Shanshan Yang: yangshanshan12@126.com

Ziqi Liu: [liuziqi9720@163.com](mailto:liuziqi9720@163.com)

*** Corresponding author:** Weiwei Gao

E-mail address: [wwgao@implad.ac.cn/wwgao411@sina.com](mailto:wwgao@implad.ac.cn/wwgao411@sina.com)

^#^Na Peng and Yanmeng Bi contributed equally to this work.

Institute of Medicinal Plant Development, Chinese Academy of Medical Science and Peking Union Medical College, Beijing, 100193, China

Tel.: +86 10 57833423; Fax: +86 10 87283423.


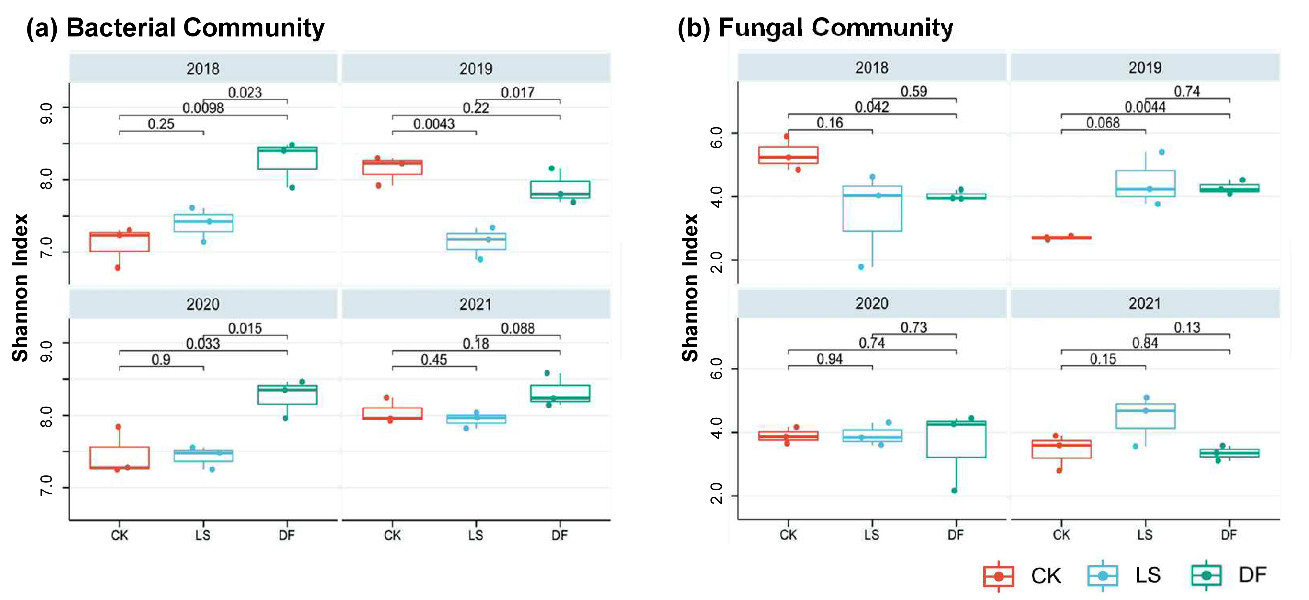


**Fig. S1.** Effect of different soil fumigant treatment (CK: without soil fumigant; LS: lime sulfur; DF: dazomet fumigation) on bacterial (a) and fungal (b) diversity from 2018 to 2021. The tops and bottoms of boxes represent the 75th percentiles and 25th percentiles, respectively. The values above the horizontal line represents a significant difference between the two groups


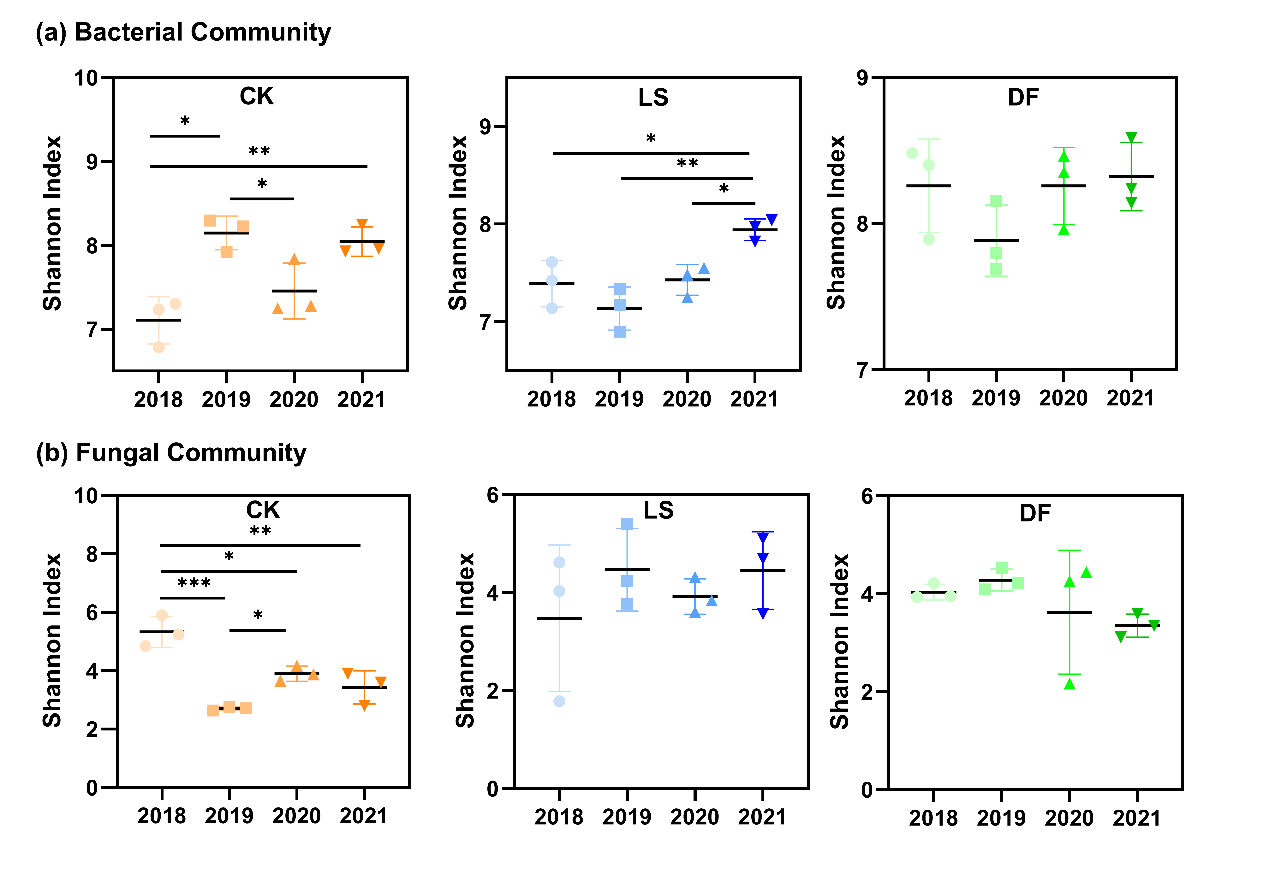


**Fig. S2.** Effect of different soil fumigant treatment (CK: without soil fumigant; LS: lime sulfur; DF: dazomet fumigation) on bacterial (a) and fungal (b) diversity from 2018 to 2021.


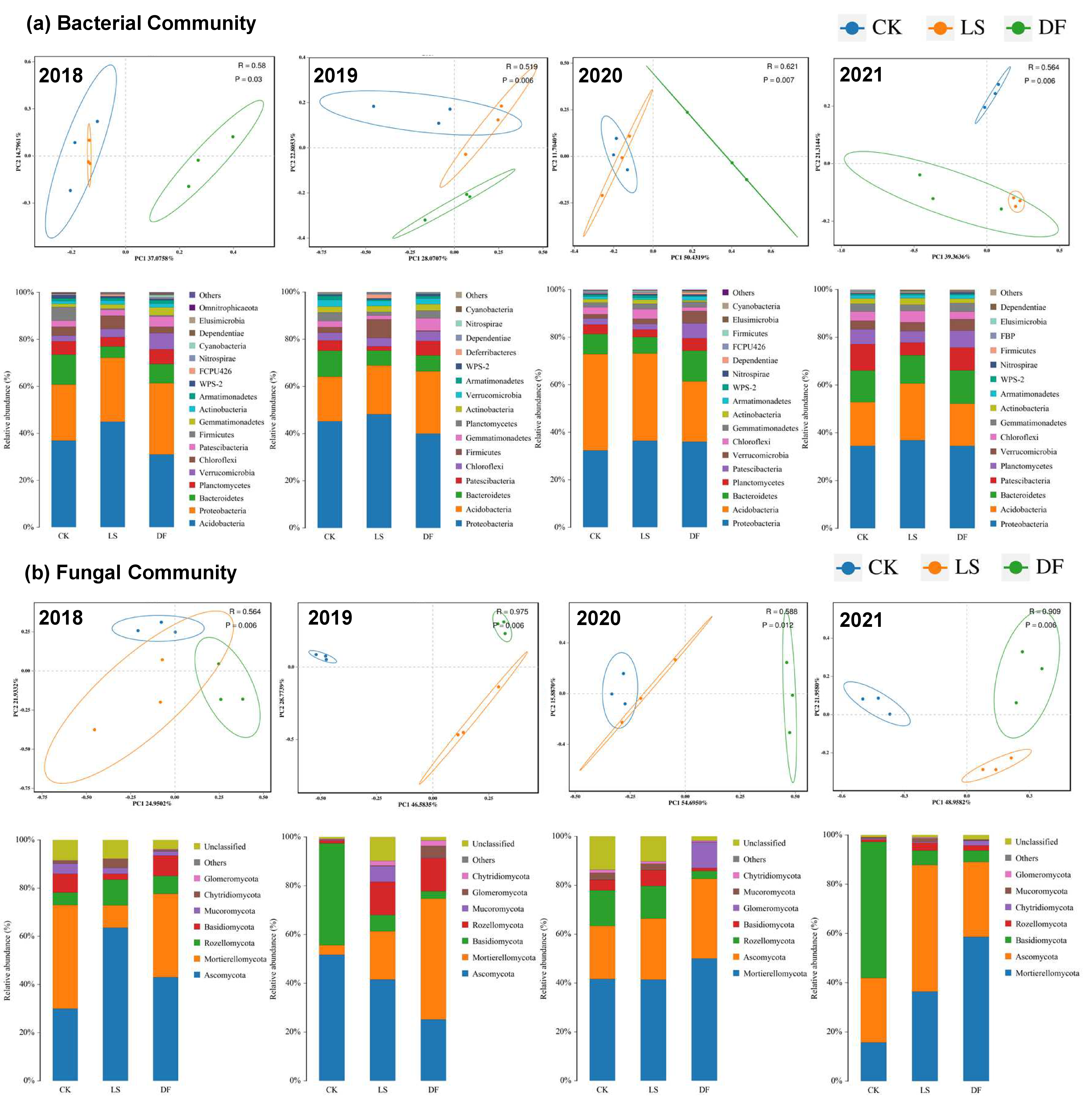


**Fig. S3.** Effect of different soil fumigant treatment (CK: without soil fumigant treatment; LS: lime sulfur treatment; DF: dazomet fumigation treatment) on bacterial and fungal community composition from 2018 to 2021. (a) The principal coordinate analysis (PCoA) based on the 16S rRNA sequences and the relative abundances of bacterial phyla. (b) The principal coordinate analysis (PCoA) based on the ITS sequences and the relative abundances of fungal phyla.


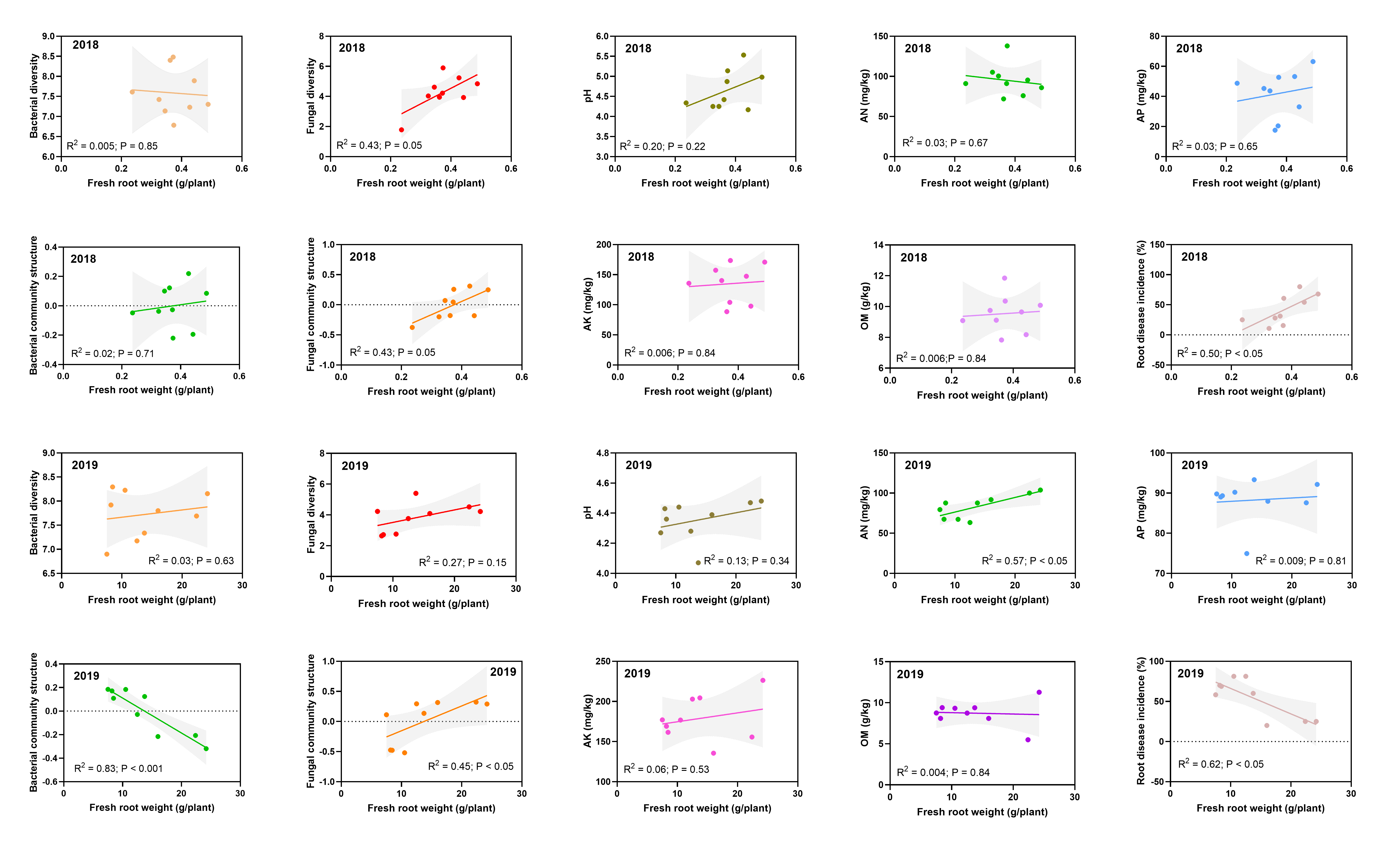


**Fig. S4** Scatterplots showing correlation between fresh root weight and bacterial diversity, bacterial community structure, fungal diversity, fungal community structure, pH, AK, AN, OM, AP, and root disease incidence from 2018 to 2019. Different maker symbols indicate significant statistical differences (*p < 0.05; **p < 0.01; ***p < 0.001) according to spearman correlation analysis.


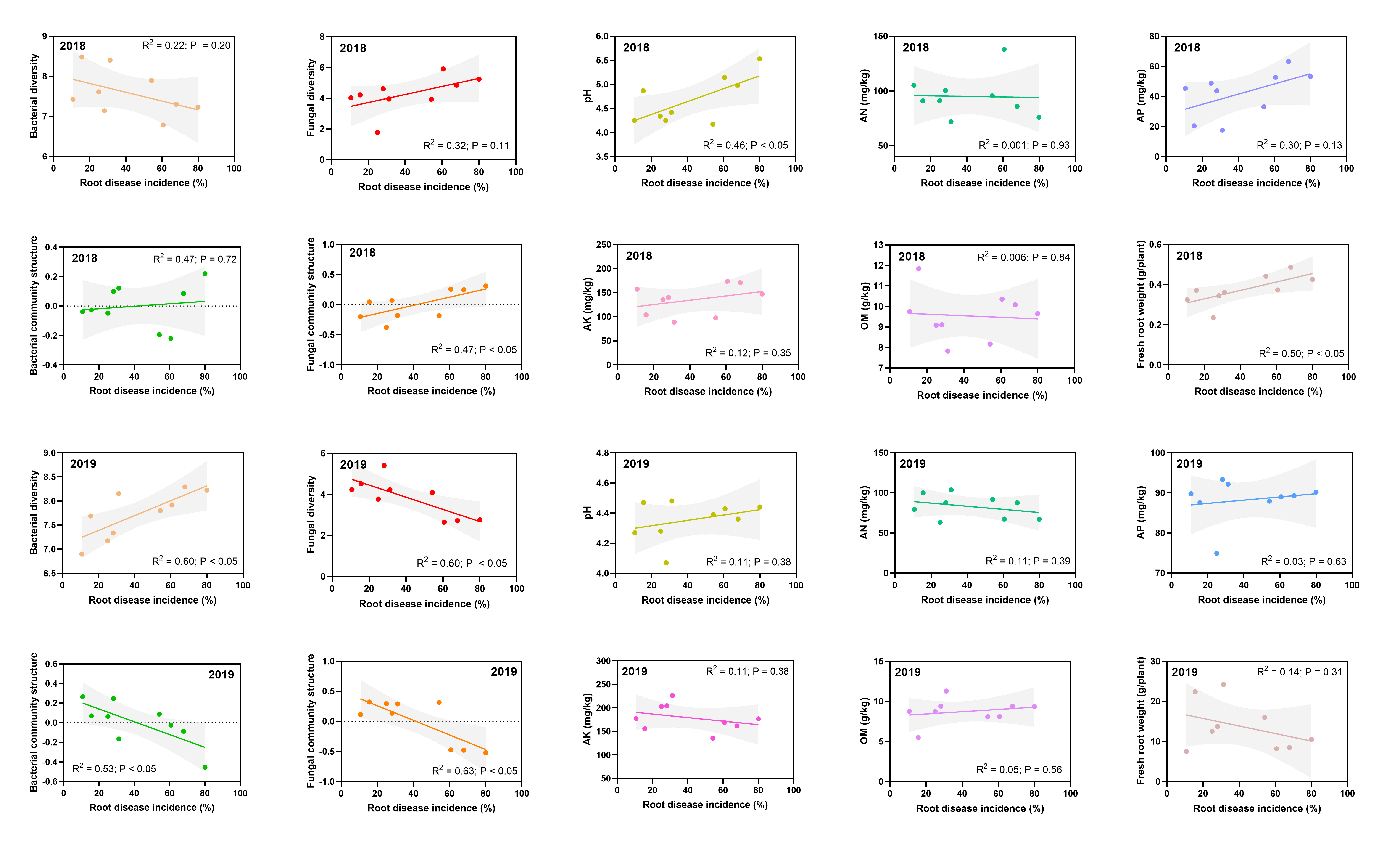


**Fig. S5** Scatterplots showing correlation between root rot disease incidence and bacterial diversity, bacterial community structure, fungal diversity, fungal community structure, pH, AK, AN, OM, AP, and fresh root weight from 2018 to 2019. Different maker symbols indicate significant statistical differences (*p < 0.05; **p < 0.01; ***p < 0.001) according to spearman correlation analysis.

**Table S1** The chemical properties of the original soil before American ginseng planting (mean ± standard error, n=3).

|  | **pH** | **AN (mg/kg)** | **AP (mg/kg)** | **AK (mg/kg)** | **OM (g/kg)** |
| --- | --- | --- | --- | --- | --- |
| CK | 4.55 ± 0.24 a | 115.07 ± 21.81 a | 51.77 ± 5.50 a | 213.49 ± 8.54 a | 11.70 ± 0.37 a |
| LS | 4.34 ± 0.11 a | 98.90 ± 9.55  a | 45.97 ± 6.10 a | 167.25 ± 43.58 a | 10.94 ± 1.60 a |
| DF | 4.64 ± 0.36 a | 110.12 ± 21.81 a | 28.41 ± 2.41 b | 184.55 ± 48.69 a | 9.93 ± 1.24 a |

**Table S2** The chemical properties of the rhizosphere soil after CK, LS, and DF treatments (mean ± standard error, n=3).

| **Soil property** |  | **2018** |  |  | **2019** |  |  | **2020** |  |  | **2021** |  |
| --- | --- | --- | --- | --- | --- | --- | --- | --- | --- | --- | --- | --- |
|  | **CK** | **LS** | **DF** | **CK** | **LS** | **DF** | **CK** | **LS** | **DF** | **CK** | **LS** | **DF** |
| **pH** | a 5.21  ± 0.28 | 4.28  ±0.05b | 4.49  ±0.35b | 4.41  ±0.04b | 4.21  ±0.12b | 4.45  ±0.05b | 4.91± 0.10a | 4.99  ±0.26a | 4.98  ±0.02a | 4.95± 0.18a | 5.02  ±0.05a | 5.03  ±0.03a |
| **AN (mg/kg)** | 99.92 ±33.33a | 98.86  ±7.15a | 86.15  ±12.47a | 74.12  ±11.71a | 76.77  ±12.40a | 98.56  ±6.16a | 69.99  ±4.76a | 67.33  ±7.07a | 75.53  ±0.07a | 59.13  ±8.20b | 61.86  ±4.67a | 78.16  ±10.15a |
| **AP (mg/kg)** | 56.36 ± 5.87a | 45.92  ±2.58b | 23.70  ±8.26c | 89.53  ±0.60d | 86.03  ±9.76d | 89.23  ±2.53d | 87.76  ±5.59d | 93.24  ±3.13d | 85.93  ±2.37d | 89.72  ±7.09d | 91.62  ±2.45d | 89.04  ±0.86d |
| **AK (mg/kg)** | 136.98±14.38a | 144.47  ±11.45a | 96.80  ±7.83b | 169.11  ±7.61c | 194.82  ±15.36d | 172.52  ±47.62ade | 125.15  ±4.06ae | 97.91  ±3.13b | 101.04  ±32.61ab | 122.09  ±22.05ab | 97.07  ±18.08ab | 136.97  ±11.98ab |
| **OM (g/kg)** | 10.03± 0.36a | 9.32  ±0.37a | 9.29  ±2.23a | 8.95  ±0.73a | 8.96  ±0.37a | 8.29  ±2.90a | 5.93  ±0.38b | 5.50  ±0.01b | 6.59  ±0.97b | 4.21  ±1.11c | 4.00  ±0.76c | 7.19  ±1.33ab |

Values are presented as means ± SD (n = 3). Differences were assessed by ANOVA and denoted as letters. AN: alkaline nitrogen, AP: available phosphorus, AK: available potassium, OM: soil organic matter
